# Auto-Thermal Reforming Using Mixed Ion-Electronic Conducting Ceramic Membranes for a Small-Scale H_2_ Production Plant

**DOI:** 10.3390/molecules20034998

**Published:** 2015-03-18

**Authors:** Vincenzo Spallina, Tommaso Melchiori, Fausto Gallucci, Martin van Sint Annaland

**Affiliations:** Chemical Process Intensification, Chemical Engineering and Chemistry, Eindhoven University of Technology, 5600MB Eindhoven, The Netherlands; E-Mails: v.spallina@tue.nl (V.S.); t.melchiori@tue.nl (T.M.); f.gallucci@tue.nl (F.G.)

**Keywords:** MIEC membranes, auto-thermal reforming, membrane reactors, syngas production, energy analysis

## Abstract

The integration of mixed ionic electronic conducting (MIEC) membranes for air separation in a small-to-medium scale unit for H_2_ production (in the range of 650–850 Nm^3^/h) via auto-thermal reforming of methane has been investigated in the present study. Membranes based on mixed ionic electronic conducting oxides such as Ba_0.5_Sr_0.5_Co_0.8_Fe_0.2_O_3-δ_ (BSCF) give sufficiently high oxygen fluxes at temperatures above 800 °C with high purity (higher than 99%). Experimental results of membrane permeation tests are presented and used for the reactor design with a detailed reactor model. The assessment of the H_2_ plant has been carried out for different operating conditions and reactor geometry and an energy analysis has been carried out with the flowsheeting software Aspen Plus, including also the turbomachines required for a proper thermal integration. A micro-gas turbine is integrated in the system in order to supply part of the electricity required in the system. The analysis of the system shows that the reforming efficiency is in the range of 62%–70% in the case where the temperature at the auto-thermal reforming membrane reactor (ATR-MR) is equal to 900 °C. When the electric consumption and the thermal export are included the efficiency of the plant approaches 74%–78%. The design of the reactor has been carried out using a reactor model linked to the Aspen flowsheet and the results show that with a larger reactor volume the performance of the system can be improved, especially because of the reduced electric consumption. From this analysis it has been found that for a production of about 790 Nm^3^/h pure H_2_, a reactor with a diameter of 1 m and length of 1.8 m with about 1500 membranes of 2 cm diameter is required.

## 1. Introduction

Currently approximately 95% of all the hydrogen used is produced from fossil fuels as primary feedstock, where technologies such as natural gas reforming and coal gasification are applied at very large scale [1]. Hydrogen’s share in the energy market is increasing with the implementation of fuel cell systems and the growing demand for zero-emission fuels. The potential of hydrogen as a future energy carrier has been reviewed by different authors [2,3], who concluded that the costs associated with building the infrastructure and with transporting hydrogen over large distances are not sustainable from an economic point of view and in particular for the transport sector. Additionally, large-scale hydrogen production plants are dependent on the market volumes and subjected to the cost evolution of the products. An attractive option is a small stationary unit for on-site hydrogen production via steam methane reforming (SMR) or methane partial oxidation (POX) [4]. Auto-thermal reforming (ATR) combines both SMR and POX so that the heat required for the endothermic reaction is supplied by using a proper amount of oxidant and no external furnace and indirect heat exchangers are required, thus making the system more compact. All these systems also require water-gas-shift reactor(s) to enhance the H_2_ yield and some form of gas purification such as a pressure swing adsorption (PSA) system. Using air as oxidant for the auto-thermal reaction leads to higher volume flow rates and a high fuel consumption due to the intrinsic dilution with N_2_. When using pure oxygen, cryogenic air separation is currently the most efficient and mature technology for producing large quantities of O_2_ (>0.75 t/h of pure O_2_) with a specific energy consumptions in the range of 160–250 kWh/t_O2_ depending on the O_2_ purity [5], but inefficient for small scale applications, where different other systems have recently been proposed based on N_2_ adsorption, molten salt absorption and polymeric membranes [6]. 

**Figure 1:**
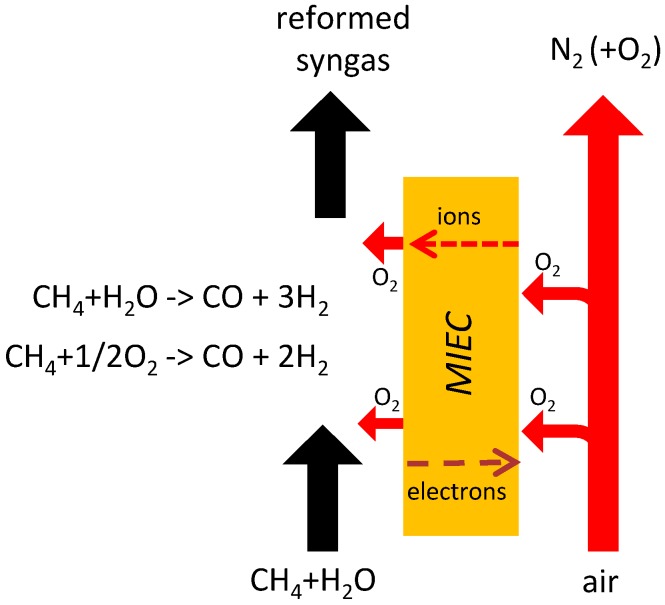
Schematic of an ATR-MR based on oxygen transport membrane.

The study of inorganic membrane (also called mixed ionic-electronic conducting membrane or MIEC) materials and processes for O_2_ production has increased rapidly in recent years due to the potential to improve the overall efficiency and environmental footprint, while reducing the cost [7,8,9,10,11] In a MIEC membrane, O_2_ permeates through the membrane after applying to the system a driving force which can be either an electrical potential gradient or a chemical potential gradient. The MIEC membrane can also be integrated in a membrane reactor in case the permeated O_2_ reacts at the permeate side, as shown in Figure 1. 

The use of MIEC membranes for integrated O_2_ separation has been studied for different applications, where most of the proposed ones relate to the production of syngas and oxy-combustion systems [12,13]. Praxair Inc. is working on the integration of oxygen transport membranes in a coal gasification unit [13] using multi-stage oxygen membrane units operated at different pressures to gradually convert the syngas into exhaust gases and separating the by-products downstream. For a natural gas fired plant for pre-combustion CO_2_ capture [14], Chiesa *et al.* [15] proposed integrating the oxygen membranes with an auto-thermal reforming unit with CO_2_ separation by amine absorption. H_2_ production using oxygen perm-selective membranes has also been studied, amongst others by Gallucci *et al.* [16,17]. They proposed a multi-stage fluidized bed membrane reactor, where oxygen membranes are used to permeate O_2_ that reacts with methane and steam to form reformed syngas in the bottom part and where H_2_ selective membranes are used in the top part of the reactor to separate pure H_2_ and simultaneously convert the remaining syngas into a CO_2_-rich stream that can be separated and stored. MIEC membranes have also been studied for the oxidative coupling of methane to produce C_2_hydrocarbons, which is a highly exothermic reaction that requires a reaction temperature of 700–900 °C and low oxygen partial pressures to achieve high C_2_ yields [18]. O_2_ membrane reactors have also been studied to control the temperature of the system to avoid problems with hot-spot formation, which may occur in conventional auto-thermal reforming reactors, where the combustion zone is separated from the catalytic zone [19].

In this paper MIEC membranes are integrated in an auto-thermal reforming system (ATR-MR) for H_2_ production at small-to-medium scale, where the ATR-MR system is fully integrated with the other components of the plant including the micro-gas turbine. Micro-gas turbines have been studied in recent years as valid alternatives to internal combustion engines in the 10–1000 kW_e_ range, which differ from the widely used commercial heavy duty gas-turbines (mostly used in large scale combined cycles) in their different size, design and performance [20,21,22,23]. To carry out plant simulations, a model has been developed in the flowsheeting software program Aspen. The Aspen flowsheet was coupled with an external executable file that via an Excel interface imports the values of the variables related to the streams entering the reactor block (such as flow rates, compositions, temperatures, *etc.*). The specific reactor model developed in Matlab solves mass and energy balance for the membrane reactor according to the specific design and returns the values of the variables at the outlet. After that, the Aspen model performs the calculations of the downstream units. A detailed analysis of the plant is discussed in the paper with a specific focus on the design of the membrane reactor. The membrane flux, membrane dimension and scale-up is based on experimental results that have been obtained with permeation tests of a Ba_0.5_Sr_0.5_Co_0.8_Fe_0.2_O_3-δ_ (BSCF) membrane, which is shortly described first. 

## 2. Membrane Testing

**Figure 2:**
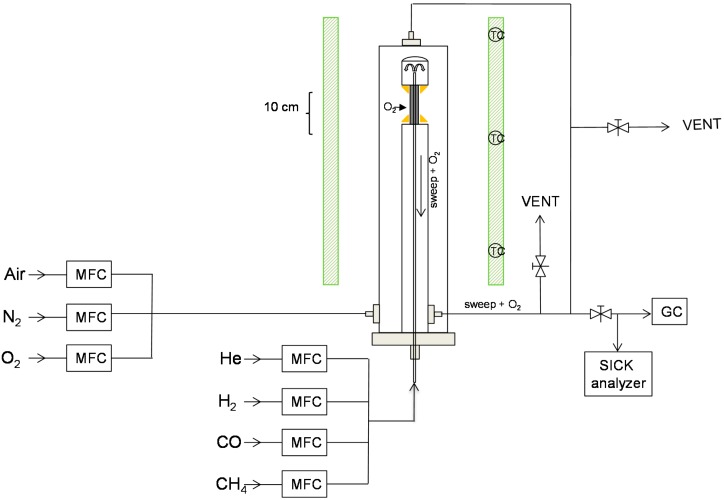
Layout of the experimental setup used for the permeation test. Membrane length: 10 cm, membrane tube outer diameter: 3.5 mm, membrane thickness: 0.5 mm.

The oxygen permeation properties of BSCF membranes were studied in a high-temperature oxygen permeation setup as shown in Figure 2.

### Description of the Experimental Setup

Air is fed from the bottom in an oven (type Eli-tube 250/26, ELicra, Nijkerk, The Netherlands) which can reach temperatures up to 1150 °C. Due to the size, the oven has three parts that are heating the entire reactor and each part is controlled by a thermocouple. The BSCF perovskite membranes were prepared by the Flemish Institute of Technological Research (VITO, Mol, Belgium). Gold paste and Schott glass were used as sealants to fix the membrane between a dense alumina tube and alumina cup [24]. A capillary was placed inside the tube (and the membrane) through which sweep gas (He or reducing gases) is fed. During the permeation experiments, a maximum heating rate of 3 °C/min was used (to prevent the sealing and membrane from cracking due to thermal stresses). Once the membrane has been activated inside the reactor, the temperatures were kept above 800 °C and below 1000 °C to retain the chemical structure of the membrane and prevent thermal stresses in the reactor. Permeation flux experiments have been conducted by adding an air flow through the shell and a sweep gas through the capillary. During non-reducing conditions, He (99.995%) was used as sweep gas.

**Figure 3:**
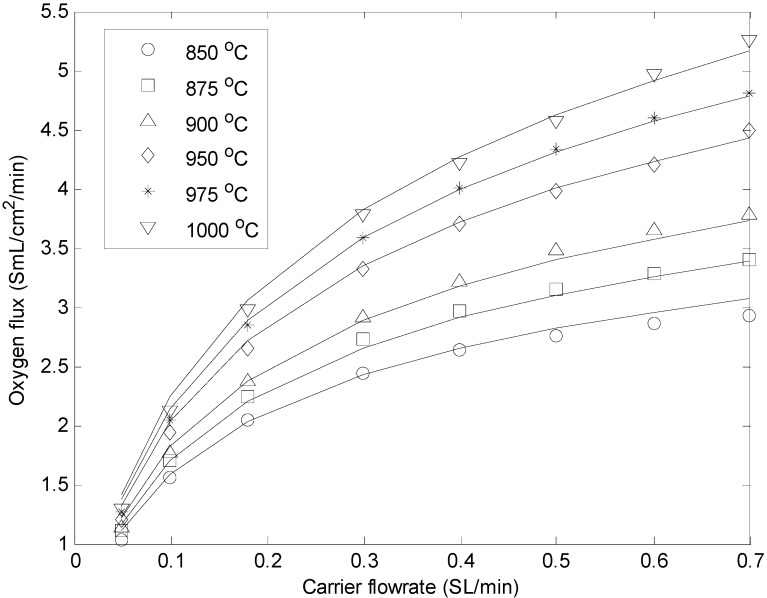
Comparison between experimental (symbols) and calculated (continuous lines) oxygen fluxes using BSCF membrane at different temperatures and carrier flow rates.

The oxygen permeation flux was measured as a function of the He flow rate (from 50 to 350 SmL/min) at different temperatures in the range of 850–1000 °C (Figure 3). The results clearly show that at higher temperatures the O_2_ permeation rate through the membrane increases, because of the higher O_2_ bulk diffusion and surface exchange kinetics. More details about these membranes can be found in [24]. As expected, increasing the sweep gas flow rate also increased the O_2_ permeation as a result of the higher partial pressure difference across the membrane. The experimental results from these permeation tests have been correlated to develop a permeation expression that has been implemented in the reactor model, as discussed in the next section.

## 3. Reactor Model

### 3.1. Description of the Model

The model describes a catalytic bed reactor in which a number of membrane tubes are immersed, and the corresponding model equations have been listed in Table 1. The catalyst is supposed to be placed in the shell side, where the partially reformed methane is fed, and where the reactions of reforming oxidation and water-gas shift take place. Inside the tubes, on the other hand, no particles are present. Air is fed here, co-current with the fuel flowing in the shell. Oxygen partially permeates through the membranes surface, and reacts with methane when it reaches the catalytic section. No back permeation of gas is assumed inside the tubes, so no reaction occurs in the air section. The model solves the chemical species balances in both the retentate (air) and permeate (fuel) side, assuming a 1D geometry for the two zones. The equations include transport by convection, production or consumption due to reaction (only in the fuel side), and production and consumption due to mass transfer from the air side to the fuel side. This term is actually present only in the equation for oxygen, because the membrane is assumed to be impermeable to all the other components because of the very high perm-selectivity measured for the BSCF membrane. The mass transfer terms are expressed as a function of the oxygen concentration difference between the bulk and the membrane surface. The mass transfer coefficients are calculated with the correlation by McCabe *et al.* [25]. 

**Table 1:** Equations for the membrane reactor model.

**Equations for the Fuel Side**	
$\frac{dc_{i}^{f}v^{f}}{dz}=r_{i}+h_{i}^{f}a^{f}\left(c_{i}^{\text{m},f}-c_{i}^{f}\right)$	(1)
$\frac{dc^{f}v^{f}}{dz}=\sum_{j=1}^{N}\left(r_{j}+h_{j}^{f}a^{f}\left(c_{j}^{\text{m},f}-c_{j}^{f}\right)\right)$	(2)
$c^{f}Cp^{f}\frac{dT^{f}}{dz}=-\sum_{j=1}^{N}\left[H_{j}(T^{f}){\text{r}}_{j}\right]-Ua^{f}\left(T^{f}-T^{a}\right)+\sum_{j=1}^{N}\left[h_{j}^{f}a^{f}\left(c_{j}^{\text{m},f}-c_{j}^{f}\right)\left(H_{j}(T^{a})-H_{j}(T^{f})\right)\right]$	(3)
$\frac{dP^{f}}{dz}=-\frac{\rho^{f}v^{f}}{d_{p}}\frac{\left(1-\varepsilon \right)}{\varepsilon^{3}}\left(\frac{150\mu^{f}\left(1-\varepsilon \right)}{\rho^{f}d_{p}}+1.75v^{f}\right)$	(4)
$r_{i}=\sum_{j=1}^{NR}\nu_{ij}R_{j}\rho_{cat}(1-\varepsilon )$	(5)
**Equations for the Air Side**	
$\frac{dc_{i}^{a}v^{a}}{dz}=-h_{i}^{a}a^{a}\left(c_{i}^{a}-c_{i}^{m,a}\right)$	(6)
$\frac{dc^{a}v^{a}}{dz}=-\sum_{j=1}^{N}\left(h_{j}^{a}a^{a}\left(c_{j}^{a}-c_{j}^{m,a}\right)\right)$	(7)
$c^{a}Cp^{a}\frac{dT^{a}}{dz}=Ua^{a}\left(T^{f}-T^{a}\right)\frac{S^{f}}{S^{a}}$	(8)
**Equations at the Membrane Interface**	
$h_{i}^{f}\left(c_{i}^{\text{m},f}-c_{i}^{f}\right)=J^{m}$	(9)
$-h_{i}^{a}\left(c_{i}^{\text{m},a}-c_{i}^{a}\right)=J^{m}\frac{S^{f}}{S^{a}}$	(10)
$k^{f}\left(T^{f}-T^{m,f}\right)=U\left(T^{f}-T^{a}\right)$	(11)
$k^{a}\left(T^{m,\text{a}}-T^{a}\right)=U\left(T^{f}-T^{a}\right)\frac{S^{f}}{S^{a}}$	(12)
**Equation for the Oxygen Flux Through the Membrane**	
$J^{m}=\frac{D_{v}k_{r}\left({\left(p_{i}^{m,a}\right)}^{n}-{\left(p_{i}^{m,f}\right)}^{n}\right)}{2\delta k_{f}{\left(p_{i}^{m,a}p_{i}^{m,f}\right)}^{n}+D_{v}\left({\left(p_{i}^{m,a}\right)}^{n}+{\left(p_{i}^{m,f}\right)}^{n}\right)}$ n = 0.5	(13)
**Heat and Mass Transfer Coefficients**	
$h_{i}^{f}=0.0096\frac{D_{i}}{d^{eq,\text{f}}}Sc_{f}^{0.346}Re_{f}^{0.913}$	(14)
$h_{i}^{a}=0.0096\frac{D_{i}}{d^{eq,\text{a}}}Sc_{a}^{0.346}Re_{a}^{0.913}$	(15)
$k^{f}=0.17\frac{\lambda^{f}}{d_{p}}{\left(\frac{Pr_{f}}{0.7}\right)}^{1/3}Re_{p}^{0.79}$	(16)
$k^{a}=0.023\frac{\lambda^{a}}{R^{m}}{\left({\text{Pr}}_{a}\;\right)}^{1/3}Re_{a}^{0.8}$	(17)
$U=\frac{1}{\frac{1}{k^{f}}+\frac{1}{k^{a}}\frac{S^{f}}{S^{a}}+\frac{R^{m}}{\lambda^{m}}\text{ln}\left(\frac{R^{m}+\delta }{R^{m}}\right)}$	(18)
**Reaction Rate Expressions**	
$R_{1}=\frac{k_{1a}p_{CH4}p_{O2}}{{\left(1+K_{CH4}^{ox}p_{CH4}+K_{O2}^{ox}p_{O2}\right)}^{2}}+\frac{k_{1b}p_{CH4}p_{O2}}{\left(1+K_{CH4}^{ox}p_{CH4}+K_{O2}^{ox}p_{O2}\right)}$	(19)
$R_{2}=\frac{k_{2}\left(p_{CH4}p_{H2O}-p_{H2}^{3}p_{CO}/K_{2}\right)}{p_{H2O}^{1.596}}$	(20)
$R_{3}=\frac{k_{3}\left(p_{CO}p_{H2O}-p_{H2}p_{CO2}/K_{3}\right)}{p_{H2O}}$	(21)

A kinetic model based on the Ni/Al_2_O_3_ catalyst was considered, including three reactions: methane total combustion, methane steam reforming and water-gas-shift reaction, as follows:

*R_1_ : CH_4_ + 2O_2_ → CO_2_ + 2H_2_O*

*R_2_ : CH_4_ + H_2_O ↔ CO + 3H_2_*

*R_3_ : CO + H_2_O ↔ CO_2_ + H_2_*

The direct reaction of partial oxidation of methane is not included in the mechanism: as a matter of fact, for the considered catalyst, and indirect route of formation of syngas is usually assumed in the literature, as shown by the study by Dissanayake *et al.* [26], followed by other authors [27,28]. According to this, steam must be formed by total combustion (when it is not directly provided), before consecutively reacting with methane to form CO and H_2_. Water gas shift is also important to determine the final ratio between the products.

The kinetics of the methane total combustion is described by the equations proposed by Trimm and Lam [29], while the methane steam reforming and water-gas-shift by the corrections by Numaguchi and Kikuchi [30]. It must be pointed out that, while Numaguchi and Kikuchi [30] actually derived their parameters for the Ni based catalyst, the model by Trimm and Lam [29] is valid for the Pt/Al_2_O_3_ system. For this reason, the equation and adsorption constants for the methane combustion were corrected for the Ni/Al_2_O_3_ catalyst, as proposed by de Smet *et al.* [28]. The corresponding kinetic parameters used in this work have been reported in Table 2. 

**Table 2:** Kinetic parameters for methane combustion, reforming and water gas shift reaction.

	Pre exponential factor	Activation energy (kJ/mol)
$k_{1a}(mol\;s^{-1}k{g_{cat}}^{-1}{\text{bar}}^{-2})$	8.11 × 10^5^	86.0
$k_{1\text{b}}(mol\;s^{-1}k{g_{cat}}^{-1}{\text{bar}}^{-2})$	6.82 × 10^5^	86.0
$k_{2}(mol\;s^{-1}k{g_{cat}}^{-1}bar^{-0.404})$	2.62 × 10^5^	106.9
k3(mol s−1kgcat−1bar −1)	2.45 × 10^2^	54.5
$K_{CH4}^{ox}(bar^{-1})$	1.26 × 10^−1^	−27.3
$K_{O2}^{ox}(bar^{-1})$	7.87 × 10^−7^	−92.8

In the catalytic section, the pressure drop over the bed is taken into account, using the Ergun equation in its differential form. A pseudo-homogeneous energy balance was also added for both the fuel and air compartments, to describe the axial temperature profiles in the tubes and shell. These equations include the source terms due to the heat of reaction, and heat exchange through the membranes. The global heat transfer coefficient was calculated considering the internal and external heat transfer resistances, as well as the heat conduction resistance through the membrane, where the heat transfer coefficients were calculated from correlations from the literature [31,32]. In the equation for the fuel side, a further source term is present that describes the effect of mixing of the reacting gas with the oxygen being transported through the membrane at a different temperature. This term is related to the enthalpy associated to the mass flux. The evaluation of the heat capacity and enthalpies of the single species is performed through the Shomate equation, with the thermodynamic parameters from the National Institute of Standards and Technology (NIST) database [33]. 

Finally, a total mass balance is considered both for the tubes and for shell side. These two equations are used to evaluate the local gas velocity that can change due to changes in the temperature, pressure, or as a result of a reaction occurring with a variation in the total number of moles, and mass transfer between the different compartments, where the ideal gas law is used as equation of state.

The oxygen membrane permeation depends on the values of the oxygen concentration at the internal and external membrane surfaces, evaluated with Equations (9) and (10), while for the oxygen permeation flux the generalized Equation (13) is used [34]. The permeability parameters were evaluated by comparing experimental results for the BSCF membrane, as shown in the next section. These parameters are also function of the membrane temperature, so the temperature profiles at the tube-shell interface are also calculated using Equations (11) and (12). Note that the equation for the oxygen permeation flux depends non-linearly on the interface oxygen partial pressures, so that the final problem to solve is a mixed differential and algebraic system (DAE). The model was implemented and compiled in Matlab.

### 3.2. Determination of Membrane Permeability Parameters

The previously described membrane reactor model was first used to estimate the values of the permeability parameters in the permeation flux expression (Equation (13)). In their studies, Xu and Thomson [34], and Hong *et al.* [35] reported values for the pre-exponential factors and activation energies to calculate *D_v_*, *k_r_* and *k_f_* for a La_0.6_Sr_0.4_Co_0.2_Fe_0.8_O_3-δ_ (LSCF)-type perovskite membrane. The experimental results reported for the BSCF membranes show higher oxygen fluxes compared to their data (as expected for this kind of membranes), and the parameters were fitted to the presented experimental data. 

In order to calculate the parameters, a modified version of the model described previously was used. The experimental data to fit were obtained by fixing the reactor temperature in each test. For this reason, the energy balance could be ignored and the temperature was fixed in the model. In addition, in the presented tests, the air flow rate was very high compared to the permeated oxygen flux, so that the oxygen fraction at the retentate side could be considered constant and only the permeate side needed to be modeled. Finally, the model was adapted to the different geometry of the experimental setup (single tube and permeate at the inner side). The model was coupled with an optimization solver that minimizes the sum of square errors, defined as the difference between the calculated and experimental oxygen fluxes for each test, by changing the values of the parameters in Equation (13). 

The results of the fitting are shown in Figure 3, and the obtained parameters are reported in Table 3. The figure shows that the agreement between experimental and calculated values is quite good with a mean relative error equal to ±2.5%.

**Table 3:** Permeability parameters obtained from the data fitting.

-	Pre exponential Factor	Activation Energy (kJ/mol)
$D_{v}(m^{2}s^{-1})$	9.823	91.8
$k_{r}(mol\;m^{-2}s^{-1})$	15.36	56.3
$k_{f}(m\;s^{-1}Pa^{-0.5})$	308.5	267.0

## 4. Description of the Plant

### 4.1. Plant Design

**Figure 4:**
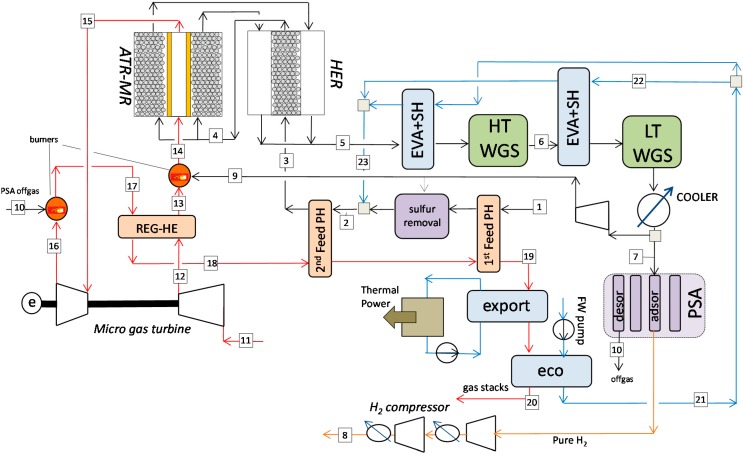
Plant design of the auto-thermal process with MIEC membranes for small-scale H_2_ production.

The schematic layout of the H_2_ production plant used in this analysis is presented in Figure 4. The main assumptions for the calculation are in Table 4. The different points (the symbol # stands for point) in Figure 4 are summarized in Table 5. The methane stream (#1) is first pre-heated up to 400 °C and mixed with steam (#23) at 400 °C. The feed is pre-heated to 600 °C and sent to the Heat Exchanger Reforming (HER). In the HER, the feed gas passes through a catalytic bed in which both gas heating and steam methane reforming take place while the hot flue gases from the ATR Membrane Reactor are passing through the tubes immersed in the catalytic reactor and are cooled by supplying the heat required for the reaction. Despite the fact expensive metal dusting resistant materials are required compared to conventional adiabatic primary reformer units, the HER has been commercialized [36] thanks to the optimal heat recovery accomplished with this system. The HER is also widely studied especially for ATR units, where the use of pre-reforming boosts the overall efficiency [37]. The syngas is fed to the ATR-MR at 650 °C (#4) partially reformed. The methane conversion into reformed syngas is enhanced by the addition of the pure oxygen which is permeating via the MIEC membranes. The temperatures of the gases (at the permeate and retentate sides) increases and the CH_4_ conversion is almost complete. The hot gasses are leaving the HER at around 700 °C and are afterwards cooled down by producing part of the required steam for the process at 400 °C (and the desired pressure). After two stages of water gas shift (WGS), the gasses are further cooled down and the H_2_O in the stream is condensed and separated. Two WGS stages at high and low temperatures are beneficial for the system because they increase the amount of H_2_ produced while reducing the heat content of the PSA-offgas. Part of the H_2_-rich stream is burnt (#9) to pre-heat the air used in the MIEC and the remaining part (#7) is fed to the PSA where pure H_2_ is produced and then compressed to the desired pressure (#8). The inlet air (#11) is compressed in an air compressor (#12) and it is pre-heated in two steps: in the first step air is heated up to 600 °C in a regenerative heat exchanger using high temperature exhausts coming from the post-combustor downstream the gas turbine (#17). This temperature is limited to about 650–700 °C when stainless steel or advanced austenitic components are used, otherwise very expensive alloy materials are required with an increase of the regenerative heat exchanger cost of 3.5 up to 5.5 times [38]. The air stream is finally heated up to the operating inlet temperature of the membranes (800–900 °C) by burning part of the H_2_-rich stream (#9). At the membranes outlet the O_2_-depleted air (#10) is expanded in the gas turbine and afterwards the stream #16 is used to burn the PSA-offgas. It should be noted that the PSA offgas could be burnt before the expansion of the gas to increase the efficiency of the gas turbine with a gain in the net power production from the micro gas turbine. However, this last solution requires a PSA-offgas compressor and a more complicated gas turbine layout, which also includes a specific cooling system to avoid excessive overheating of the blades. The O_2_-depleted air is finally cooled down (#19) by pre-heating the fuel feed, and used for combined heat production of intermediate pressure (20 bar) steam at 220 °C and to supply the required heat for the economizer that is used to heat up the H_2_O for process (#21). 

### 4.2. Main Assumptions and Methodology

The analysis of the plant and the ATR-MR design has been studied for different configurations, where the main assumptions used for the calculations have been reported in Table 4 (the main assumptions are taken from [39]). The operating pressure of the ATR-MR has been varied from 5 bar to 20 bar. Although conventional micro-gas turbines are typically operated with lower compression ratios (from 2 up to 10 for the high pressure system), a higher compression ratio has been selected in order to investigate a wider range of operating conditions for the reactor. For the micro gas turbine no cooling system has been considered and the main assumptions are taken from [40] assuming that the compressor is a centrifugal turbomachine and the turbine is a radial inflow expander. 

With the studied configuration, the amount of steam produced is sufficient to meet a steam-to-carbon-ratio (S/C) in the range of 2–2.8. This number is relatively high for ATR reactors that usually operated with a S/C in the range of 1.5 to 2. However, due to the high amount of heat available in the system the high temperature (HT) syngas coolers have been used for the production of steam for the process. The H_2_ separation efficiency of the PSA has been assumed equal to 80% (the main assumption is that this efficiency is not function of the operating pressure). 

**Table 4:** List of assumptions used in the analysis.

Main Assumptions	
Fuel feedstock composition (% Vol.)	100% CH_4_
LHV_NG_ (MJ/kg)	50 MJ/kg
Ambient air conditions	1 bar, 15 °C
Water feed conditions	1 bar, 15 °C
Air composition (%vol)	79% N_2_; 21% O_2_
Process conditions	
Pre-reforming inlet temperature, °C	650
Maximum reforming temperature, °C	900–1200 °C
Reforming pressure, bar	5–20
steam-to-carbon ratio	2–3
HT-WGS inlet temperature °C	400
LT-WGS inlet temperature, °C	250
Pressure drops, % of inlet pressure	1
Heat Exchangers	
ΔT_min_ gas-gas	20
ΔT_min_ gas-liquid	10
Pressure drops, % of inlet pressure	2
H_2_ compressor and PSA	
PSA H_2_ Separation purity	100%
PSA H_2_ separation efficiency*	80%
H_2_ separation process, bar	5–20
Number of intercooled compression stages	depending on the permeate pressure
Final H_2_ pressure for plant export, bar	20
H_2_ outlet temperature, °C	30
Polytropic efficiency for compression stages,%	80%
pump/compressors mech-electric efficiency, %	95%
Gas Turbine	
Air compressor isentropic efficiency	75%
Gas expander isentropic efficiency	70%
Electric mechanic efficiency	95%
Steam cycle parameters	
pressure drops economizers, % of inlet pressure	25%
pressure drops superheaters, % of inlet pressure	8%
Max. steam temperature, °C	400
steam export at 20 bar	saturated
pump hydraulic efficiency	80%
pumps mech-electric efficiency	94%

The overall plant has been simulated with Aspen Plus 7.3 and the membrane reactor mass and energy balances have been simultaneously solved with the dedicated 1D model implemented in Matlab as described before. In this way, after fixing the geometry of the membrane reactor and the main operating conditions, simulation is able to return the overall plant performance and reactor characteristics, such as the permeated O_2_ flow rate, gas velocity, pressure drops, gas composition and temperature profiles along the membrane reactors. This characteristic of the model permits to simultaneously perform the process and reactor design and optimization. 

**Table 5:** Thermodynamic conditions of the streams in Figure 4.

Stream	T	p	m	N	N*LHV_mol_	Composition (% vol.)							LHV
#p	°C	bar	kg/h	kmol/h	MW	CH_4_	CO	CO_2_	H_2_	H_2_O	N_2_	O_2_	MJ/kmol
#1	15.0	10.0	252.0	15.7	3.50	1.000	0.000	0.000	0.000	0.000	0.000	0.000	802.3
#2	400.0	10.0	934.4	53.6	3.50	0.293	0.000	0.000	0.000	0.707	0.000	0.000	235.2
#3	600.0	10.0	934.4	53.6	3.50	0.293	0.000	0.000	0.000	0.707	0.000	0.000	235.2
#4	650.0	10.0	934.4	66.8	3.84	0.136	0.031	0.068	0.366	0.399	0.000	0.000	207.0
#5	700.0	9.8	1157.3	85.1	3.45	0.000	0.133	0.051	0.444	0.370	0.000	0.001	146.1
#6	433.0	9.7	1157.3	85.0	3.34	0.000	0.034	0.151	0.542	0.273	0.000	0.000	141.7
#7	35.0	9.2	709.4	58.3	3.03	0.000	0.010	0.236	0.754	0.000	0.000	0.000	186.9
#8	30.0	20.0	70.9	35.2	2.38	0.000	0.000	0.000	1.000	0.000	0.000	0.000	244.0
#9	44.5	10.1	69.0	5.7	0.29	0.000	0.010	0.236	0.754	0.000	0.000	0.000	186.9
#10	35.0	1.1	638.5	23.1	0.64	0.000	0.025	0.594	0.381	0.000	0.000	0.000	100.0
#11	15.0	1.0	2642.8	91.6	0.00	0.000	0.000	0.000	0.000	0.000	0.790	0.210	0.00
#12	367.3	10.3	2642.8	91.6	0.00	0.000	0.000	0.000	0.000	0.000	0.790	0.210	0.00
#13	600.0	10.3	2642.8	91.6	0.00	0.000	0.000	0.000	0.000	0.000	0.790	0.210	0.00
#14	900.0	10.0	2711.8	95.1	0.00	0.000	0.000	0.015	0.000	0.045	0.761	0.179	0.00
#15	863.7	10.0	2489.0	88.1	0.00	0.000	0.000	0.016	0.000	0.049	0.821	0.115	0.00
#16	522.9	1.1	2489.0	88.1	0.00	0.000	0.000	0.016	0.000	0.049	0.821	0.115	0.00
#17	1000.2	1.0	3127.5	106.6	0.00	0.000	0.000	0.147	0.000	0.123	0.679	0.051	0.00
#18	835.2	1.0	3127.5	106.6	0.00	0.000	0.000	0.147	0.000	0.123	0.679	0.051	0.00
#19	618.4	1.0	3127.5	106.6	0.00	0.000	0.000	0.147	0.000	0.123	0.679	0.051	0.00
#20	107.1	1.0	3127.5	106.6	0.00	0.000	0.000	0.147	0.000	0.123	0.679	0.051	0.00
#21	15.0	1.0	682.4	37.9	0.00	0.000	0.000	0.000	0.000	1.000	0.000	0.000	0.00
#22	175.0	11.0	213.3	11.8	0.00	0.000	0.000	0.000	0.000	1.000	0.000	0.000	0.00
#23	400	10	682.4	37.9	0.00	0.000	0.000	0.000	0.000	1.000	0.000	0.000	0.00

Since the reforming efficiency, given by Equation (22), only takes into account the amount of produced H_2_, a different index has been used in order to compare the plant performance including also the electricity and heat produced/consumed as proposed by Martinez *et al.* [41]. To consider the contribution of the electricity and the heat flows exchanged with the environment, the equivalent natural gas (NG) thermal input has been calculated according to the equation (23), in which the reference thermal efficiency (*η_th_* equal to 90%) corresponds to the conventional industrial boiler used for steam production and the electric efficiency represents the typical performance of a NG-fired combined cycle (*η_el_* assumed equal to 58.1%):

(22) $$\eta_{ref}=\frac{{\dot{m}}_{H_{2},pure}LHV_{H_{2}}}{{\dot{m}}_{GN}LHV_{GN}}$$

(23) $$\eta_{ref,eq}=\frac{{\dot{m}}_{H_{2},pure}LHV_{H_{2}}}{{\dot{m}}_{GN,eq}LHV_{GN}}\;\text{where}\;{\dot{m}}_{GN,eq}={\dot{m}}_{GN}-\frac{E_{el}}{\eta_{el}LHV_{GN}}-\frac{Q_{th}}{\eta_{th}LHV_{GN}}$$

## 5. Results and Discussion

### 5.1. Detail of the Plant Flowsheet

The design of the system has been optimized by varying the main parameters that affect the plant performance. As far as the membrane reactor is concerned, the sensitivity analysis has been focused on the size of the reactor (reactor diameter and length) and the number of membranes. The optimization is carried out in order to maximize the equivalent H_2_ efficiency. The analysis of the results also includes the main effects on the plant design, which are subsequently further discussed. The detailed mass balance of the reference case is reported in Table 5 and it is referring to an ATR-MR module operated at 10 bar, fuel fed at 650 °C after pre-reforming using a mixture of methane and H_2_O (S/C = 2.4) and (depleted) air fed at 900 °C with an O_2_ concentration of 17.9% after the pre-burner. At the permeate side, pre-reformed syngas is produced at 1127 °C: the CH_4_ is completely converted, and the CO content is 13.3%. The overall CO conversion is 94.4%, where after the first WGS stage 75% of the CO is already converted and after the low temperature Water Gas Shift (LT-WGS) a final CO concentration of 1% (dry basis) is obtained. The amount of H_2_-rich syngas used in a pre-combustor to pre-heat air from 600 °C up to 900 °C is 8.9% of the total syngas produced, while the main stream is sent to the PSA unit. At the retentate side, the O_2_-depleted air is released at 863 °C with 11.5% of O_2_ and expanded to 1.05 bar. The gas leaving the expander is heated up to about 1000 °C after the post-combustor that is used to convert the PSA-offgas coming from the desorption beds. 

The exhaust streams are cooled down to 620 °C by pre-heating the fuel feed streams and after that they are used to produce saturated steam at 20 bar (0.19 kg/s). The exhaust gases are finally cooled down by pre-heating the process water and released to the stack at 107 °C. The total amount of pure H_2_ produced is 0.02 kg/s, which corresponds to about 790 Nm^3^/h. Looking at the quality of conversion of the syngas (in terms of energy), it is important to note that the energy of the fuel increases after the pre-reforming (from 3.5 to 3.84 MW), as expected after an endothermic reaction, and slightly decreases at the outlet of the ATR-MR, explained by the combination of the endothermic reaction of SMR and the reaction with O_2_ where an oxygen-to-carbon ratio (O/C) of 0.89 is achieved, indicating that a large part of the chemical energy is converted into heat inside the system. After that, the energy of the fuel decreases because of the WGS reaction and, after the PSA the amount of energy in the pure H_2_ stream is 2.38 MW which corresponds to a reforming efficiency of 68%. 

The presence of HER which works at high temperature (650 °C) impacts significantly with the overall efficiency of the system. More than 57% of the CH_4_ at the feed side is converted in the pre-reforming reactor and the remaining providing about 65% of the H_2_ produced in the reforming reactor (and 53% of the total amount of H_2_ produced). 

In terms of electricity requirements, the net power consumption is 28.34 kW_e_. The low Turbine Inlet Temperature (TIT) which results from the ATR-MR model significantly reduces the efficiency of the thermodynamic cycle and the overall power production is almost zero (+1.4 kW) since the power produced from the expander is balanced by the compressor consumption. The power requirement to bring the pure H_2_ from the PSA separation pressure (9.2 bar) up to 20 bar is 28.5 kW, while the auxiliary consumptions of the syngas fan and feedwater pump are about 0.8 kW. The reactor has been designed with a shell diameter of 1 m and a shell (and membrane) length of 1.8 m with 1500 membranes with a diameter of 2 cm. With this design about 60% of the reactor volume is occupied by the membranes and the feasibility of this design must be further verified from an engineering point of view.

The results show that the reforming efficiency is approaching 70% (the equivalent reforming efficiency is about 76%–77%) which is slightly lower than the system operated with fired tubular reforming as presented in [41]. however this configuration is designed for small-to-medium scale applications and therefore the operating conditions, such as the steam production and the turbomachines are not as efficient as in the case of a large scale H_2_ plant.

### 5.2. Sensitivity Analysis

A sensitivity analysis has been carried out on the effect of the operating pressure and the temperature of the oxidant at the inlet and the results are summarized in the Table 6. An additional analysis includes the effect of the reactor design on the performance of the plant. The O_2_ concentration at the retentate side has been kept constant (in the range of 11.4%–11.9%) in order to ensure enough O_2_ to incinerate the PSA-offgas in the post-combustor. 

The micro-gas turbine power is strictly dependent on the gas temperature at the ATR-MR retentate side. In order to increase the temperature, the reactor size has been increased. Increasing the reactor volume (about 10 times and 20 times), the outlet temperature at the retentate side increases from 863.7 °C to 925 °C and consequently the power required from the exterior decreases from −28.3 kW to −12.4 kW. Looking at the energy balance, this increase is caused by a higher power for the turbine. A larger reactor volume also allows for a larger available membrane surface area. Moreover, the inlet conditions of the reactor—such as S/C, gas composition and temperature—remain substantially the same, but due to the different amount of heat transfer in the system the amount of O_2_ permeation changes since it strongly depends on the membrane surface temperature. This issue will be further discussed in the next section.

The sensitivity analysis has also been carried out concerning the operating pressure of the system. Increasing the operating pressure of the ATR-MR the following effects on the plant performance can be deduced:

◾ The fuel conversion at the pre-reforming stage is lower and therefore more heat of reaction is required in the ATR-MR, so that more oxygen has to be permeated from the air and therefore more air has to be used since the same O_2_ volume fraction in the depleted air exhaust was specified for this analysis. The resulting O/C at the ATR-MR increases from 0.8 to 0.94.
◾ Due to the lower heat required at the pre-reforming stage the amount of steam produced for the process increases by increasing the S/C (from 2.2 to 2.9), because more heat is available from the exhaust gas cooling. However, the combination of higher pressure (which reduces the reforming conversion) and higher S/C (which improves the methane conversion) in the pre-reforming section the performance has slightly decreased.
◾ The amount of pure H_2_ decreases (with a reforming efficiency drop from 70.5% to 66.6%) due to the higher O/C used which increases the CH_4_ conversion by the methane partial oxidation instead of steam methane reforming which is particularly high at the pre-reforming stage.
◾ In terms of electricity production, the micro-gas turbine (micro-GT) net electricity production decreases because a low compressor ratio (*β_compressor_*, in the range of 5) is optimum to maximize the efficiency of the thermodynamic cycle operated with such TIT; the electricity consumption for the H_2_ compressor is significantly lower (from 56.2 kW at 5 bar to 3.3 kW at 20 bar) due to the higher H_2_ separation pressure; it must be pointed out that in this analysis the efficiency of the PSA in terms of H_2_ purity has been kept constant, however, it is known that at low pressure the purity of H_2_ decreases unless some systems adopting vacuum PSA are applied [42]. The overall power consumption increases by increasing the operating pressure.
◾ At higher pressure the amount of heat for export increases due to more heat available in the plant at lower temperature which also results from a lower thermal input (from CH_4_) converted into H_2_ and/or electricity.
◾ The equivalent reforming efficiency slightly decreases from 77% to 74%.

Another sensitivity analysis has been carried out by increasing the O/C ratio in the system in order to have the streams at the reactor outlet at higher temperature. The main consequence of an increased O/C ratio is a lower amount of pure H_2_ produced with a drop in the reforming efficiency of about 5.4 percentage points. Due to the higher temperature resulting from the ATR-MR and especially at the retentate side, the overall electricity consumption significantly drops in particular because the micro-GT net electricity increases with about 24 kW_e_ with a corresponding decrease in the total power consumption from 28.6 to 4.3 kW_e_. At higher O/C ratios also the thermal power for export significantly increases (more than 20%) due to the higher temperature of the gasses. In terms of equivalent reforming efficiency, the increase in the O/C reduces the performance of the system from 76% to 73.6%, however, the decreases in the H_2_ production is partly balanced by the lower power consumption and thermal production. At higher O/C ratios the membrane area of the ATR-MR increases with 35% compared to the reference case due to higher amount of O_2_ to be separated.

Finally, the inlet temperature of the oxidant has been considered to be 800 °C. Compared to the same case operated with air fed at 900 °C it can be seen that a higher O/C ratio is required in order to reach similar outlet temperatures. The O_2_ volume fraction at the feed side of the ATR-MR is 18.9% instead of 17.9%. In this case, less fuel is required after the WGS reactors to be used in the first burner (and therefore the consumption of the syngas fan is about 30% less). The high amount of heat available in the syngas coolers (also because the syngas flow rate is higher) allows the system to work with higher S/C ratios (in the range of 3–4). Less power is consumed for H_2_ compression since less pure H_2_ is produced (the reforming efficiency drops from 62%–63% to 59%–60%) with a gain in the electricity export of about 8 kW_e_. The reactor volume (small case) is higher when operating at 800 °C in order to ensure a high O_2_ conversion at the permeate side and the membrane area is also higher especially because of (i) the average temperature of the membrane is lower, especially at the entrance of the reactor, with a correspondingly lower O_2_ permeation flux, while (ii) a higher amount of O_2_ that has to be separated.

### 5.3. Reactor Design 

To further elucidate the effect of the reactor design and behavior on the plant performance, in this section the axial concentration and temperature profiles in the ATR-MR are discussed in more detail. It must be noticed that the membrane diameter adopted in this analysis is 2 cm instead of 3.5 mm as for the experimental tests: the reason we used a bigger diameter is related to the required upscaling in case of small/medium size application.

**Table 6:** Results of the sensitivity analysis.

Summary of Performance											
Oxidant Inlet Temperature, °C	900									800	
Case	10 bar			5 bar		20 bar		10 bar high O/C		10 bar high O/C	
	Reference Small	Medium	Big	Small	Medium	Small	Medium	Small	Medium	Small	Medium
Reactor design											
N membrane	1500	1400	750	1370	1350	1500	1430	1520	1480	1750	1600
Diameter, m	1	2	2	1.1	2	1	2	1.2	2	1.5	2
Length, m	1.8	5	10	2.1	5	1.8	5	2.4	5	3	5
Main parameters											
pure H_2_, kg/h	70.93	71.72	71.47	73.44	73.51	69.35	69.34	65.26	65.11	61.59	63.40
air flow rate, kg/h	2642.8	2642.8	2642.8	2360.6	2360.6	2875.2	2875.2	3257.2	3257.2	3444.0	3444.0
O_2_ separated, kg/h	222.88	213.24	215.73	201.85	199.08	235.86	234.16	265.13	268.36	332.39	313.79
O_2_ ret fraction, %vol.	11.5%	11.8%	11.7%	11.4%	11.5%	11.6%	11.7%	11.7%	11.6%	11.4%	11.9%
steam for process, kg/h	682.43	643.11	642.20	634.80	581.02	841.97	780.06	722.89	799.08	1101.30	926.44
O/C	0.89	0.85	0.86	0.80	0.79	0.94	0.93	1.05	1.07	1.32	1.25
S/C	2.41	2.27	2.27	2.24	2.05	2.98	2.76	2.55	2.82	3.89	3.27
T_out ret_, °C	863.7	899.9	924.9	871.1	915.1	863.9	908.4	928.6	994.0	978.4	1012.0
T_out perm_, °C	1127.0	1065.3	1054.1	1103.3	1092.7	1107.2	1064.8	1238.4	1205.8	1231.7	1243.4
Energy Balance											
compressor, kW_e_	−272.58	−272.58	−272.58	−153.58	−153.58	−429.02	−429.02	−335.95	−335.95	−355.22	−355.22
turbine, kW_e_	273.61	283.61	289.70	184.33	191.82	360.49	375.59	358.87	379.08	384.54	397.20
net micro-GT, kW_e_	1.03	11.02	17.12	30.75	38.25	−68.53	−53.43	22.91	43.12	29.32	41.97
H_2_ compressor, kW_e_	−28.57	−28.89	−28.79	−56.19	−56.24	−3.31	−3.31	−26.28	−26.22	−24.81	−25.54
FW pump, kW_e_	−0.30	−0.29	−0.29	−0.13	−0.12	−0.79	−0.73	−0.32	−0.36	−0.49	−0.41
syngas fan, kW_e_	−0.49	−0.49	−0.49	−0.53	−0.53	−0.66	−0.66	−0.62	−0.62	−0.44	−0.43
net electric power, kW_e_	−28.34	−18.64	−12.45	−26.09	−18.64	−73.28	−58.13	−4.31	15.92	3.58	15.59
Q_export_, kW_th_	376.08	364.25	367.31	280.93	308.92	430.70	448.98	477.15	422.48	363.79	397.99
reforming efficiency	68.1%	68.9%	68.6%	70.5%	70.6%	66.6%	66.6%	62.7%	62.5%	59.1%	60.9%
equivalent reforming efficiency	76.1%	77.1%	77.1%	76.3%	77.5%	74.0%	75.1%	73.6%	72.8%	67.0%	70.3%
A_mem_/A_mem,ref_	1.00	2.59	2.78	1.07	2.50	1.00	2.65	1.35	2.74	1.94	2.96
O_2_ flux, mol_O2_/(s-m^2^)	0.0114	0.0042	0.0040	0.0097	0.0041	0.0121	0.0045	0.0100	0.0050	0.0087	0.0054

**Figure 5:**
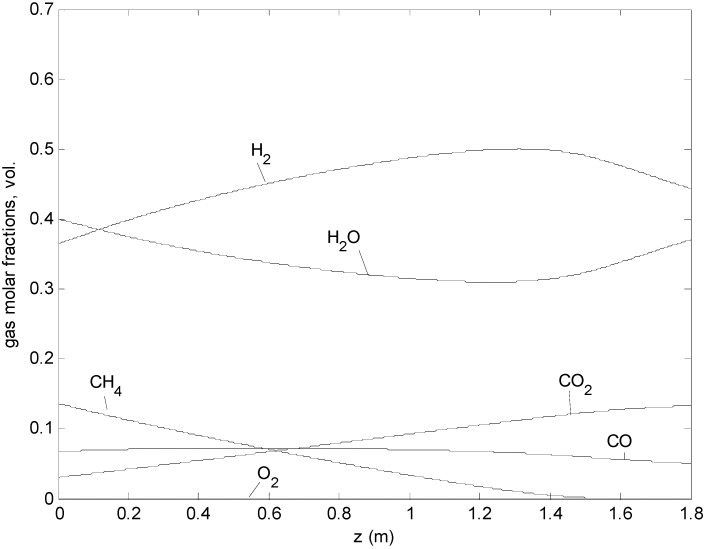
Axial mole fraction profiles at the permeate side of the ATR-MR (reference case).

**Figure 6:**
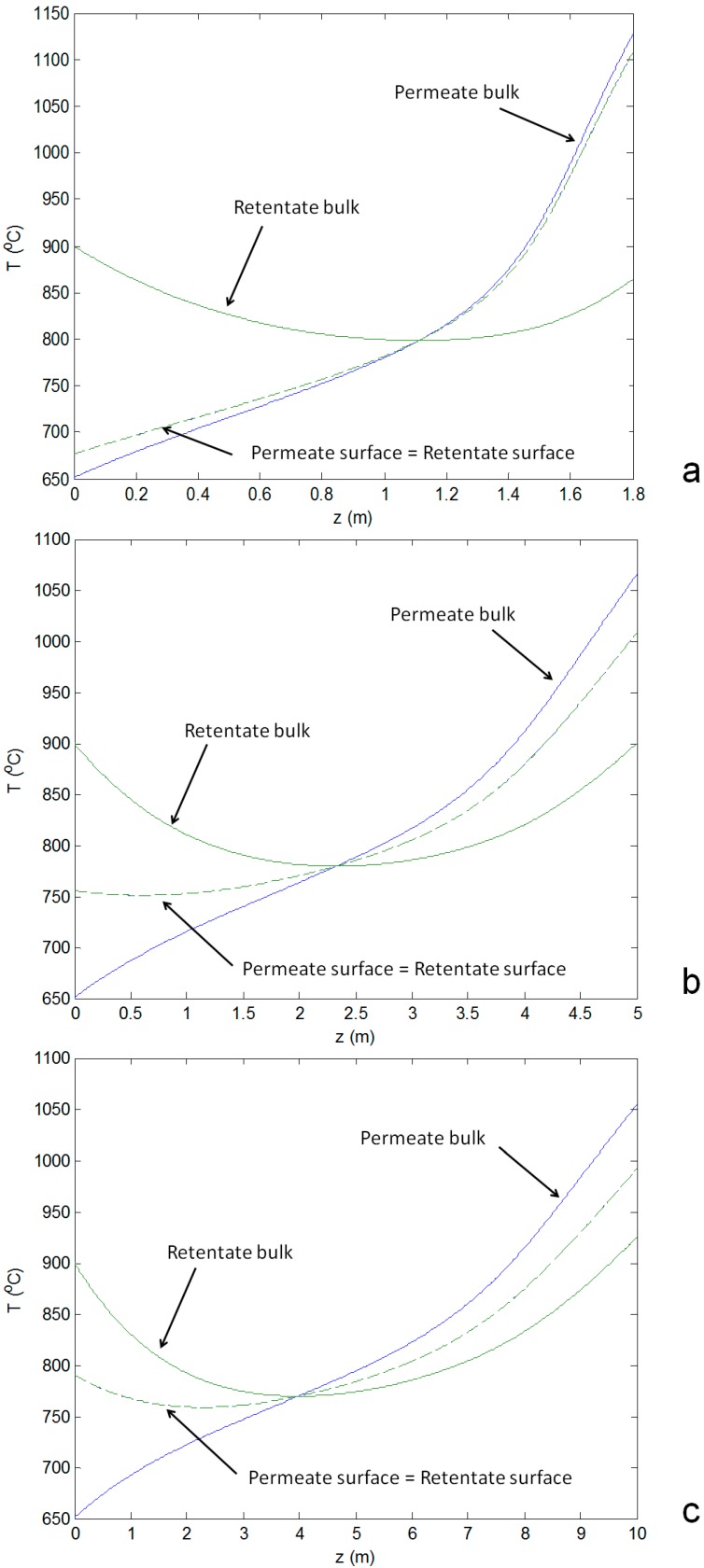
Axial temperature profiles of the gas streams and the membrane surface: (**a**) small size reactor; (**b**) medium size; (**c**) big size reactor. Continuous lines are related to the bulk phase and dashed lines are related to the membrane surface at retentate (green) and permeate (blue) side.

For the reference case the gas composition at the permeate side is depicted in Figure 5: the CH_4_ conversion proceeds along the reactor length via SMR by reacting with H_2_O which decreases in the first part of the reactor, and POX by reacting with the permeated O_2_ which is always at very low concentration (below 10^−3^ in mole fraction). H_2_ is mostly produced in the first part of the reactor while, after the CH_4_ conversion is approaching 100%, H_2_ and CO are consumed. Based on the proposed kinetics mechanism the O_2_ reacts only with the CH_4_ (R1) which is virtually instantaneously produced (due to the methanation and reverse WGS reactions which are at the equilibrium) and burnt by reacting with the remaining O_2_, thus overall resulting in H_2_ and CO combustion. In the same way, two different temperature rise velocities along the reactor can be distinguished, in particular, the first part where the cooling effect of the SMR is prominent and, the last part where the combustion reactions prevail, as shown in Figure 6. 

Based on the gas species profile, after the CH_4_ is completely converted (around 1.4 m) the maximum H_2_ production is achieved. However, as shown in Figure 6a, the final temperature of the gas at the permeate and retentate sides would be 800 °C and 900 °C respectively. The lower gas temperatures at the reactor outlet will have two main effects on the thermal balance of the plant: (i) the gas turbine will be operated with a lower TIT, reducing the electrical efficiency and therefore increasing the overall electric consumption; (ii) a lower permeate gas temperature will reduce the heat available at pre-reforming unit, and (iii) less steam will be produced for the process which affect the CH_4_ conversion in the pre-reforming section.

A temperature cross-over can be observed for all cases (Figure 6): at the beginning, the temperature of the air side is higher than the temperature at the fuel side, so the air is partially cooled down. At the same time, oxygen starts permeating, combustion occurs, and this creates a further temperature increase in the fuel side, until its temperature exceeds the temperature at the retentate side. From this point on, the retentate is heated up. Both temperatures increase because the thermal contribution of combustion is always prevailing in the last section of the reactor. The temperatures at the surface of the permeate and retentate sides coincide (and cannot be distinguished in Figure 6) because the membrane thickness is small (500 μm) and therefore the heat transfer resistance is negligible compared to the others. When the methane concentration is low, methanation occurs (reverse reforming), and the produced CH_4_ is immediately burned by the oxygen permeating from the membrane tubes. In this sense, oxygen is shifting the equilibrium of steam reforming towards the reagents. The global effect is the oxidation of syngas to steam and carbon dioxide. With respect to Figure 5 and Figure 6a, for instance, at z = 1.6 m, where the CO (13.4%) and H_2_ (49.1%) concentrations are decreasing and the temperature is increasing, the methane concentration in the catalytic area is almost zero (0.08%). In these conditions the reverse reforming reaction rate (methanation) is equal to 2 mol/m^3^/s. The reverse water gas shift rate is 5.7 mol/m^3^/s. Additionally, the methane combustion rate is 2.9 mol/m^3^/s, according to the kinetic mechanism considered in this paper [28]. The combination of these reaction rates results in an overall decrease in the CO and H_2_ concentrations and heat production.

It is important to note that the axial temperature profile does not show a typical drop in the temperature at the beginning of the reactor which occurs in case of co-feeding CH_4_ and H_2_O. In fact, within this configuration, 40% of the CH_4_ has been already converted in the HER system and therefore the heat management of the ATR-MR system is much less complicated, especially in view of the oxygen transport membrane (OTM) which requires high temperatures to achieve acceptable O_2_ permeation.

**Figure 7:**
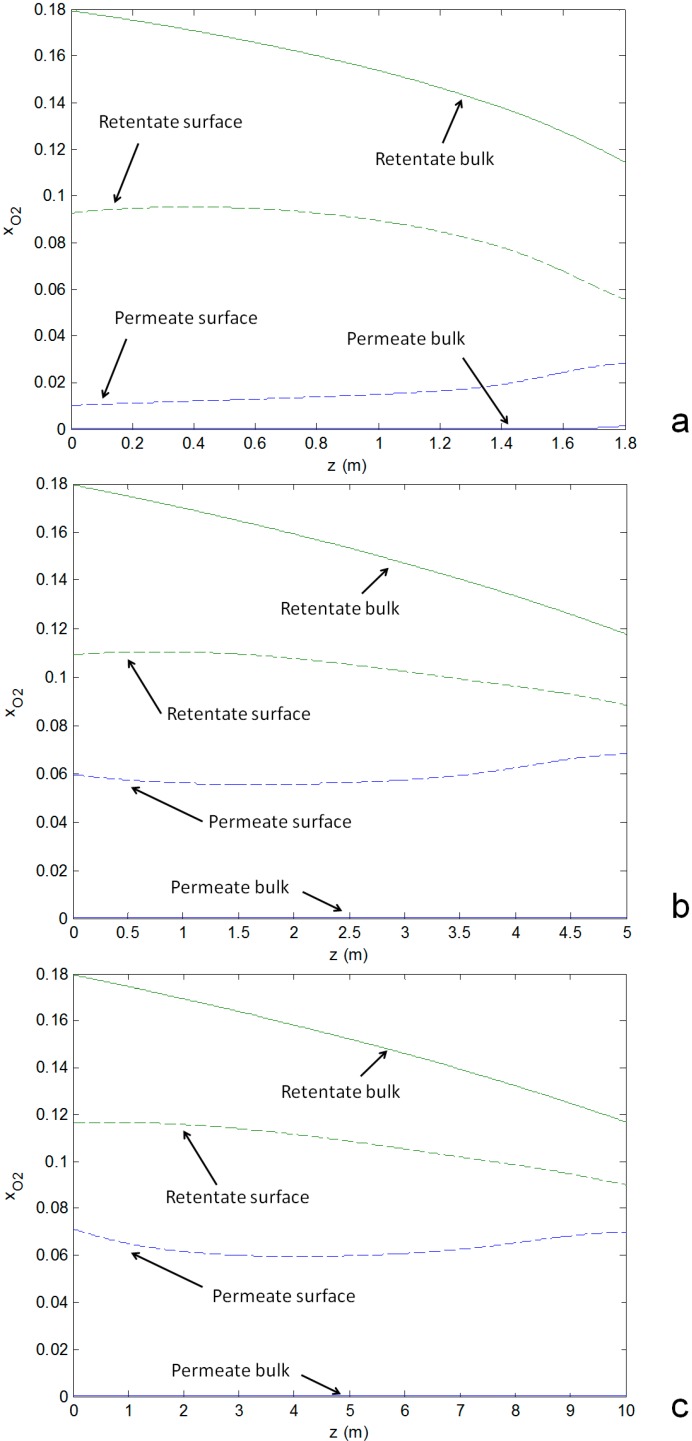
Oxygen volume fraction profiles along the reactor of the gas streams and the membrane surface. (**a**) small size reactor; (**b**) medium size; (**c**) big size reactor. Continuous lines are related to the bulk phase and dashed lines are related to the membrane surface at retentate (green) and permeate (blue) side.

A comparison between the performance of the ATR-MR with different reactor sizes shows that:

◾ For small reactors the axial temperature profile at the membrane surface follows closely the permeate bulk temperature profile. When increasing the reactor size, the axial temperature profile at the membrane surface tends to be closer to the average temperature of both reactor compartments (permeate and retentate), as a result of the increased residence time which increases the heat transfer from both sides of the reactor (Figure 6a *vs.* Figure 6b,c). Consequently, the resulting reactor outlet temperatures of the streams are also closer when increasing the reactor size.
◾ The heat transfer coefficient at the shell side (the permeate side) is higher in the case of a small reactor size due to the higher gas velocity which reduces the difference between the temperature of the bulk flow and the membrane surface at the permeate side.
◾ Despite the larger membrane area by increasing the reactor volume and length, the permeated oxygen is substantially the same because of mass transfer limitations prevailing in the shell (permeate side): in the small reactor the difference of the O_2_ concentration between the retentate and permeate side is higher (Figure 7a) than in the other cases (Figure 7b,c) which corresponds to a higher O_2_ flux. In fact, at the permeate side the gas velocity decreases by increasing the reactor diameter and therefore the mass transfer coefficient decreases because of the decreased Reynolds number. A small difference occurs between the medium and big size of the reactor due to the similarities in the reactor geometry and total membrane area.

The system operated with a small reactor shows that the O_2_ conversion at the reactor outlet is not complete (Figure 7a), because the rate of the O_2_ consumption depends on the amount of catalyst in the reactor which is apparently insufficient for the small reactor design. In this respect, a proper design of the system should consider a different shell and membrane length: in the last part no oxygen should permeate but the gas should be in contact with catalyst in order to enhance the conversion approaching almost to the thermodynamic equilibrium. From a thermal point of view, this configuration would also be beneficial for the outlet temperature of the retentate, because of the increased contact time so that part of the produced heat can be exchanged with the O_2_ depleted air to increase the TIT of the micro-GT.

This analysis shows the influence of the reactor design on cycle performance. It is worthy to note that from an engineering point of view, increasing the reactor size would increase the cost of the plant due to the material required, the catalyst and membrane area. In case the cost of the ATR-MR impacts significantly to the overall capital cost then the improvement in the overall efficiency (1–3 percentage points depending on the operating conditions as shown in Table 6) would not be beneficial for the economics of the system. For a more precise insight into this issue, a separate techno-economic analysis of this plant should be performed considering the interaction between the size of reactor and duty of the downstream units.

## 6. Conclusions 

The integration of an ATR-MR with oxygen transport membranes in a small-to-medium scale H_2_ production plant has been studied in this work. The OTM’s have been tested in the lab, exhibiting a relatively high O_2_ permeability, and the main transport parameters for the oxygen permeation through the BSCF membrane have been determined from the experimental data. A detailed membrane reactor model has been developed to describe the axial gas compositions and temperature profiles on the retentate and permeate sides and has subsequently been used for the design of the membrane reactor and the calculation of the required membrane area. The results show that in the configuration with a bigger reactor the heat transfer is increased, thereby decreasing the temperature difference between the permeate and retentate sides with a corresponding improvement in the electric performance and reduced thermal gradients along the reactor. In terms of mass transfer, the O_2_ flux is reduced when using a bigger reactor, but the conversion of the permeated O_2_ increases according to the kinetic model. The assessment of the plant performance and a sensitivity analysis of the main parameters has been carried out with Aspen Plus software and the reactor model has been included so that the results of the model were simultaneously updated during the simulation. The results show that the reforming efficiency is approaching 70% (the equivalent reforming efficiency is about 76%–77%). The combination of the ATR-MR with HER allows to recover efficiently the heat of the syngas and reduces the heat duty for the reforming, however, the cost of the entire system must be verified. From an economic point of view also the reactor size must be carefully considered: in particular, due to the difference in the electricity import required for different reactor sizes, it could be beneficial for the system to have a bigger reactor (with the associated capital cost) and less operating costs for auxiliaries and in particular for the H_2_ compressor. Another important aspect is the possibility to couple the system with a CO_2_ capture and utilization. In fact CO_2_-rich stream from the PSA-offgas can be fully oxidized in a dedicated OTM module and separated afterwards by water condensation instead of using chemical absorption processes which are high capital cost units. However, the advantages in terms of performance and the economic feasibility of an H_2_ production plant with reduced CO_2_ emissions at small medium scale must be verified.

## Nomenclature and Abbreviations

$a^{f}$, $a^{a}$: Membrane surface/reactor volume ratio for the fuel and air zone (m^2^/m^3^)
$c^{f}$,$c^{a}$: Total molar concentration in the fuel and air zone (mol/m^3^)
$c_{i}^{f}$, $c_{i}^{a}$: Molar concentration of the i-th species in the fuel and air zone (mol/m^3^)
$c_{i}^{\text{m},f}$, $c_{i}^{\text{m},\text{a}}$: Molar concentration of the i-th species at the membrane surface in the fuel and air zone (mol/m^3^)
$Cp^{f}$, $Cp^{a}$: Heat capacity in the fuel and air zone (J/mol/K)
$D_{i}$: Diffusion coefficient of the i-th species in the gas mixture (m^2^/s)
$d^{eq,\text{a}}$: Diameter of the membrane tubes (m)
$d^{eq,\text{f}}$: Equivalent diameter of the fuel zone (m)
$d_{p}$: Particle diameter (m)
$H_{i}$: Specific enthalpy of the i-th species (J/mol)
$h_{i}^{f}$,$h_{i}^{a}$: Wall-bulk mass transfer coefficient of the i-th species in the fuel and air zone (m/s)
$J^{m}$: Oxygen flux through the membrane (mol/m^2^/s)
$k^{f}$,$k^{a}$: Wall-bulk heat transfer coefficient in the fuel and air zone (W/m^2^/K)
$k_{i}$: Kinetic constant of the i-th reaction
$K_{i}$: Equilibrium constant of the i-th reaction
$P^{f}$: Total pressure in the fuel zone (Pa)
$p_{i}$: Partial pressure of the i-th species (Pa)
$p_{i}^{m,f}$,$p_{i}^{m,\text{a}}$: Partial pressure of the i-th species at the membrane surface in the fuel and air zone (Pa)
$Pr^{f}$,$Pr^{a}$: Prandtl number
$R_{i}$: Rate of the i-th reaction (mol/kg_cat_/s)
$R^{m}$: Membrane radius (m)
$r_{i}$: Production rate of the i-th species (mol/m^3^/s)
$Re^{f}$,$Re^{a}$: Reynolds number $\left(\frac{\rho vd^{eq}}{\mu }\right)$
${\text{Re}}_{p}$: Reynolds number related to the catalyst particle $\left(\frac{\rho^{f}v^{f}d_{p}}{\mu^{f}}\right)$
$S^{f}$,$S^{a}$: Membrane outer and inner surface (m^2^)
$Sc^{f}$,$Sc^{a}$: Schmidt number
$T^{f}$,$T^{a}$: Absolute temperature in the fuel and air zone (K)
$T^{m,f}$,$T^{m,\text{a}}$: Absolute temperature at the membrane surface in the fuel and air zone (K)
U: Global heat transfer coefficient (W/m^2^/K)
$v^{f}$,$v^{a}$: Gas velocity in the fuel and air zone (m/s)
z: Axial reactor coordinate (m)
β: Compressor ratio
δ: Membrane thickness (m)
ε: Bed porosity
$\lambda^{f}$,$\lambda^{a}$: Thermal conductivity of the gas in the fuel and air zone (W/m/K)
$\lambda^{m}$: Thermal conductivity of the membrane (W/m/K)
$\nu_{ij}$: Stoichiometric coefficient of the i-th species in the j-th reaction
$\mu^{f}$: Gas viscosity in the fuel area (Pa·s)
$\rho_{cat}$: Catalyst particle density (kg/m^3^)
$\rho^{f}$: Gas density in the fuel area (kg/m^3^)

## Abbreviations

ATR: Auto-thermal reforming
BSCF: Barium Strontium Cobalt Iron perovskites
DAE: differential and algebraic system
ECO: Economizer
EVA: Evaporator
GC: Gas chromatograph
HER: Heat exchanger reforming
LHV: Lower heating value
LP/HP: Low/High pressure
LT/HT: Low/High temperature
LSCF: Lanthanum Strontium Cobalt Iron perovskites
MFC: Mass flow controller
MIEC: Mixed ionic electronic conducting
MR: Membrane reactor
NIST: National Institute of Standards and Technology
NG: Natural gas
O/C: oxygen-to-carbon ratio
OTM: Oxygen transport membrane
POX: Methane partial oxidation
PSA: Pressure swing adsorption
REG: Regenerative
S/C: steam-to-carbon ratio
SH: Superheater
SMR: Steam methane reforming
TC: Thermocouple
TIT: Turbine inlet temperature
WGS: Water gas shift
